# The Complementary Relationship Between Echocardiography and Multi-Slice Spiral CT Coronary Angiography in the Diagnosis of Coronary Artery Thrombosis in Children With Kawasaki Disease

**DOI:** 10.3389/fped.2021.670887

**Published:** 2021-06-30

**Authors:** Yun-ming Xu, Yan-qiu Chu, Xue-mei Li, Ce Wang, Quan-mei Ma, Xiao-na Yu, Xian-yi Yu, Rui Chen, Yan-lin Xing, Xue-xin Yu, Le Sun, Xiao-zhe Cui, Hong Wang

**Affiliations:** ^1^Department of Pediatrics, Shengjing Hospital of China Medical University, Shenyang, China; ^2^Department of Radiology, Shengjing Hospital of China Medical University, Shenyang, China; ^3^Department of Ultrasound, Shengjing Hospital of China Medical University, Shenyang, China

**Keywords:** children, Kawasaki disease, coronary artery lesion, thrombosis, echocardiography, CTCA

## Abstract

**Aim:** To compare the diagnostic values by using transthoracic echocardiography (ECHO) and multi-slice spiral CT coronary angiography (CTCA) for identifying coronary artery thrombosis in children with Kawasaki disease (KD).

**Methods:** Total 97 KD children with coronary artery dilation complications in our hospital from June 2012 to December 2020 were included in the study. CTCA and ECHO were performed after over 1 month of illness.

**Results:** Coronary artery thrombosis was found in 14 out of 97 patients. Among them, 10 were identified as positive by CTCA, 9 were identified as positive by ECHO, and 5 were identified as positive by both CTCA and ECHO.

**Conclusion:** Both CTCA and ECHO can be used to diagnose coronary artery thrombosis. ECHO has advantage in identifying low-density thrombus, and CTCA is better for the clot in distal coronary artery. They can complement each other.

## Introduction

Kawasaki disease (KD) was first reported by Mr. Kawasaki in 1967. It is an acute febrile eruptive disease characterized by systemic vasculitis, which occurs in children under 5 years old (1), and the most frequent complication in the cardiovascular system is coronary artery lesions (CAL). At present, KD has surpassed rheumatic fever and became the most common acquired heart disease in children in developed countries (2). The probability of progressing to coronary artery disease is 15–25% in patients without systematic treatment (3). Coronary artery aneurysm with thrombosis is the most dangerous among various complications of KD in sub-acute and convalescent. Without timely treatment, it can cause acute myocardial infarction (4–6), ischemic cardiomyopathy, and even sudden death. For patients who survived, most of them will be challenged with low quality of life and shortened life expectancy (7). Methods of monitoring coronary thrombosis include percutaneous echocardiography (ECHO), multi-slice spiral CT coronary angiography (CTCA), like selective coronary angiography performed in adults, and cardiac contrast-enhanced MR (8). In this study, we are the first comparing the advantages and limitations of ECHO and CTCA in the identifying thrombosis in coronary artery in KD patients.

## Methods and Materials

### Patients

A total of 256 KD children were diagnosed with coronary artery dilatation from Dec 2010 to Mar 2020, and they were treated in the pediatric cardiovascular ward of Shengjing hospital, China Medical University. (1) In all, 97 out of 256 cases went through ECHO and CTCA in the same period time 1 month after illness. Data for these 97 patients were retrospectively analyzed. Among them, 68 were males (70.1%) and the average age was 4.9 years old (3 months to 13 years old). (2) In 159 of 256 patients, 132 of them whose CAL were recovered within 1 month of illness and CTC was not performed; 13 of them had severe allergies and were not scheduled for CTCA. CTCA tests failed in the rest 14 of 159 patients due to inadequate sedation or heart rate exceeding 90 beats per minute.

#### Method of Examination

##### ECHO

Philips IE33, EPIC-7C color Doppler echocardiography and S12-4 probe were used. The patients were examined in a quiet state. Patients who were not cooperative were treated with rectal administration of 5% chloral hydrate 1 ml/kg (maximum dose 30 ml) in advance. The left main coronary artery (LM), left anterior descending branch (LAD), left circumflex branch (LCX), and right coronary artery (RCA) were investigated. ECHO was performed by the sonographer specializing in the cardiovascular system.

##### CTCA

Philips Iqon spectral CT and Toshiba Aquilion One CT machines were used. Patients with heart rates over 90 bpm were treated with propranolol 1 mg/kg (maximum dose 20 mg) 1 h before the examination. For those whose heart rates that continued to be over 90 bpm, metoprolol 0.5 mg/kg (maximum dose 25 mg) would be administrated sublingually. For those who were uncooperative with the examination, hibernation mixture (chlorpromazine 1 mg/kg and promethazine 1 mg/kg, maximum dose of 25 mg each) was administrated intramuscular and 5% chloral hydrate 1 ml/kg (maximum dose of 30 ml) was rectal administrated. The CT three-dimensional reconstruction of coronary artery was performed in the supine position with a slice thickness of 1.0 mm at an interval of 1.0 mm. The non-ionic contrast agent Iohexol 350 was injected intravenously through a high-pressure syringe at a rate of 2.5 ml/s, and then spiral scanning was performed. The best cardiac cycle was selected for three-dimensional reconstruction, and the coronary artery was analyzed. Both ECHO and CTCA were performed within 3 days. CTCA was performed in patients who had one of the following conditions: (1) CAL occurred over 1 month before medicine was withdrawn; (2) thrombosis was identified in ECHO; (3) giant CAA identified every year or every 2 years.

#### Diagnostic Criteria

The diagnosis of KD/incomplete Kawasaki disease (IKD) met the criteria of the Kawasaki Disease Research Committee of Japanese Ministry of health and welfare in 2002 (9) and the guidelines issued by American Heart Association (AHA) in 2004 (10). Coronary dilatation met the diagnostic criteria from the Ministry of health and welfare of Japan (11) and from new statement (12) issued by the AHA in 2017.

##### Outcome Measurement

The diameter of coronary artery in these patients were measured by both ECHO and CTCA methods, which was listed by LM, LAD, LCX, and RCA segments. Calcification and thrombosis in each coronary artery aneurysm (CAA) were defined by ECHO and CTCA.

##### Statistical Analysis

SPSS 22.0 statistical software was used for statistical processing. The normal distribution of collected data was presented by χ^2^ ±*s*. Student's *t*-test was used for comparison between the two groups. The skew distribution of collected data was presented by median (m) or interquartile interval (P25 negative P75). The count data was presented in percentage (%). The chi-square test showed that *p* < 0.05 was statistically significant. KM curve analysis between LVEF ≥45% in groups with thrombosis or not.

## Results

### General Information

The imaging data of 97 children with KD were retrospectively analyzed. Positive coronary artery thrombosis was identified in 14 out of 97 patients: 9 of 14 (64.29%) by ECHO, 10 of 14 (71.43%) by CTCA, and 5 of 14 by both ECHO and CTCA. The sensitivity of CTCA was slightly higher than that of ECHO for KD with right coronary artery aneurysm (See Table 1).

**Table 1:** The comparison of examination by ECHO and CTCA in KD patients with thrombosis.

**KD**	***n***	**Male (%)**	**Age**	**LM**		**LAD**		**LCX**		**RCA**	
				**ECHO**	**CTCA**	**ECHO**	**CTCA**	**ECHO**	**CTCA**	**ECHO**	**CTCA**
Thrombosis	14	11 (78.6)	6.1 ± 1.6	3	0	4	4	0	1	3	7
*X*^2^				3.360		0.000		0.000		2.489	
*p*				0.222		1.000		1.000		0.115	

### Case Analysis of Coronary Artery Thrombosis

**Figure 1:**
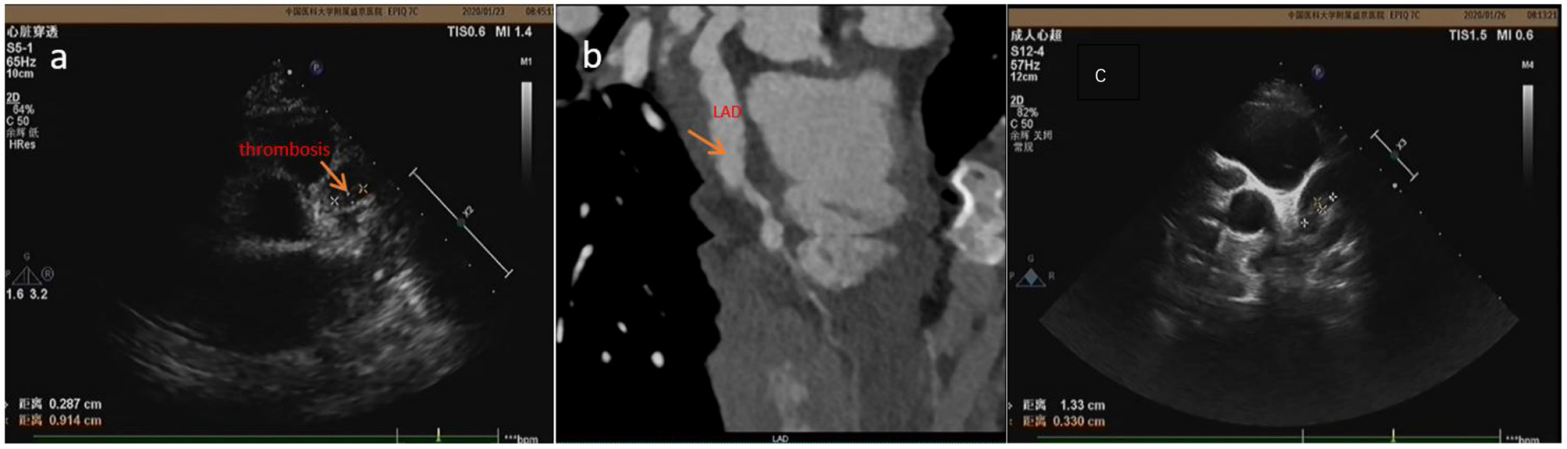
The thrombosis was detected by ECHO at 19 days **(a)** and 22 days **(c)** of illness in case 1 but wasn't detected by CTCA **(b)** at 18 days of illness.

**Figure 2:**
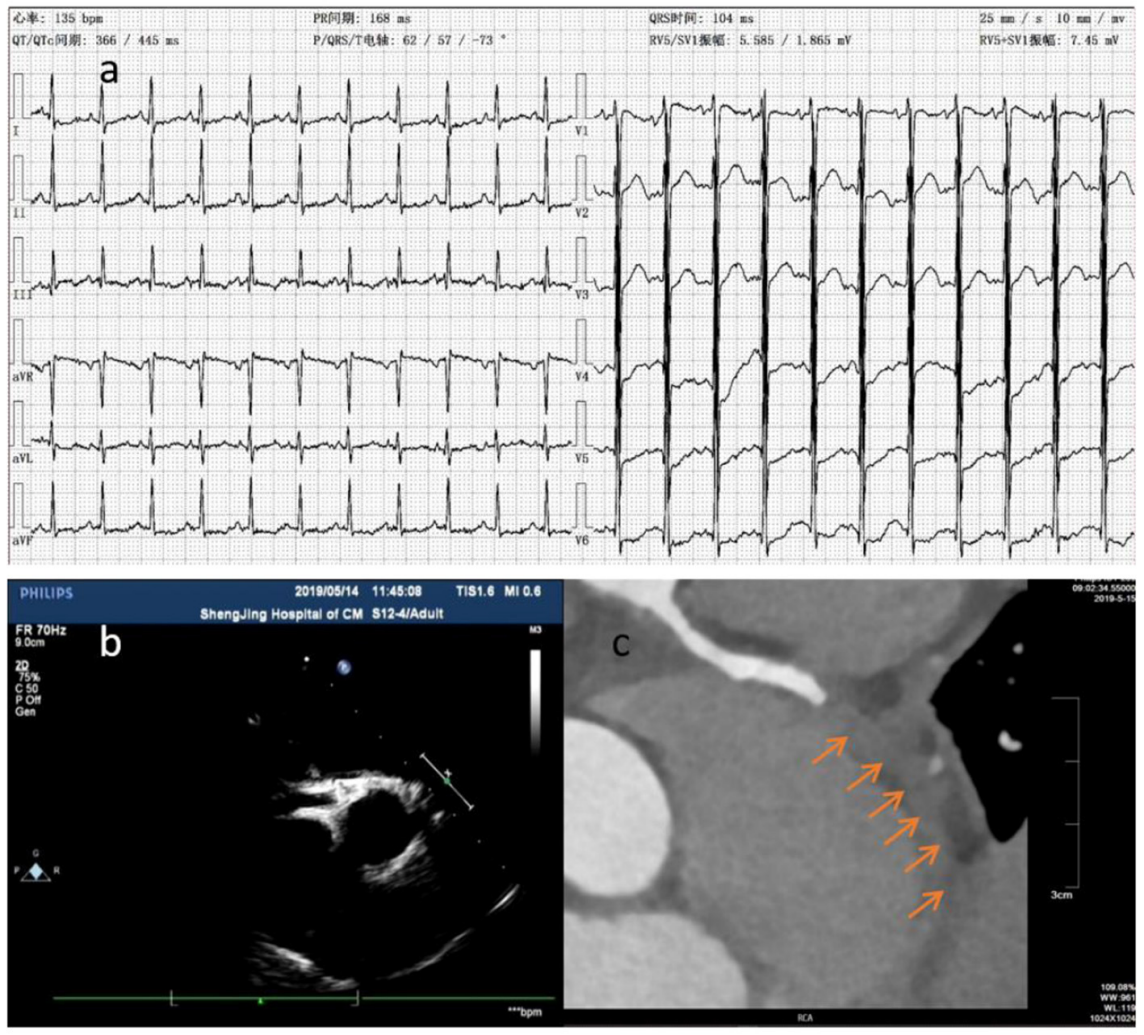
The ECG showed downward ST segment and flat T wave **(a)**. Thrombosis (arrow) was detected at distal RCA by CTCA **(c)** in case 2 at about 6.5 months of illness, whereas it wasn't detected by ECHO 2 days before and 1 day after **(b)** CTCA.

There were three cases of LM, four cases of LAD, one case of LCX, and nine cases of RCA. Diagnosis of coronary artery thrombosis was missed in four cases by CTCA, including mural thrombosis in three cases (LAD in one case, RCA in two cases) and low-density LM in one case (Figure 1). In case 5, CTCA detected thrombosis in LAD and RCA, whereas ECHO only detected thrombosis in LAD. ECHO missed distal thrombosis in RCA in five cases, and one of them (case 2) had RCA occlusion (Figure 2).

**Figure 3:**
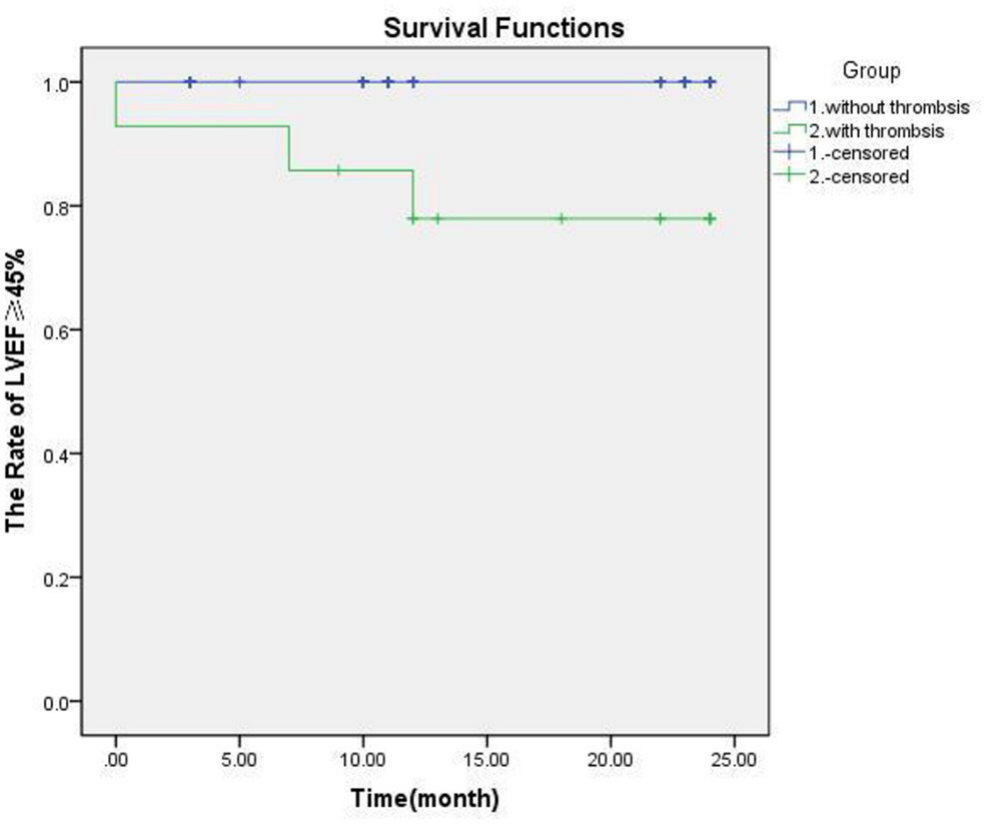
The legend needs modification. The effect of thrombosis on myocardial function and survival in patients with Kawasaki disease. There was significant difference of prognosis in groups with thrombosis or not (*p* = 0.000).

Coronary aneurysms have little effect on the blood supply to the myocardium, and the occurrence of ischemic cardiomyopathy depends on whether there is stenosis at both ends of the CAA and whether the blood flow is blocked by thrombus in the CAA. The central thrombus in CAA has the greatest impact on myocardial blood supply. The prognosis of patients with LVEF <45% was poor (the case 3 patient passed away). K-M curve analysis showed that the difference was significant (*p* < 0.001; Figure 3) (See Table 2).

**Table 2:** The location of coronary artery thrombosis identified.

**Case**	**Age(y)**	**Gender**	**ECHO**					**CTCA**				
			**LM (mm) (*Z* score)**	**LAD (mm) (*Z* score)**	**LCX (mm)**	**RCA (mm) (*Z* score)**	**Exam time (days after diagnosis)**	**LM (mm) (*Z* score)**	**LAD (mm) (*Z* score)**	**LCX (mm)**	**RCA (mm) (*Z* score)**	**Exam time (days after diagnosis)**
1	6	M	3.6–7 (1.97–9.81)	11.0 (22.17) (thrombus 3^*^9)	5.1	5.0–8.3 (5.65–12.96)	9		Middle 6.1 (9.94)	6.3	10.7–5.0 (18.28–6.31)	10
2	8	M	4.2 (3.80)	3.5 (3.83)	3.9	3.1–4.7 (1.80–6.13)	224			Stenosis of opening, proximal dilated 3.4	Middle and distal aneurysms were occlusion by thrombus	225
3	3	M	6.3 (10.57)	25^*^24 (64.99–62.20) (1–2 mm thrombus in anterior wall)		3–3.4 (2.64–3.65)	180	18			Proximal dilated	182
4	8	M	3.0 (0.66)	6.2 (10.3) (patchy thrombus)	2.6	2.6 (0.39)	2,418		Proximal aneurysm formation, possibly peri-aneurysmal thrombus			2,419
5	5	M		11.1–12.3 (21.71–24.70) thrombus		11–15 (21.43–31.08)	489		Proximal giant aneurysm 18^*^30 (43.49–75.77) (thrombus)	opening in aneurysm	Multiple giant aneurysms, bigger one 16^*^26 (33.49–57.59) (thrombus)	492
6	6	M	3.4–4.7 (1.19–4.12)	11.0–11.8 (21.42–23.37) thrombus	2.5	7.5–9.2 (10.65–14.32)	73	3.2	16.2 (34.09) multiple aneurysm (massive thrombus)	10.6^*^15 multiple aneurysm (few thrombus).	13.5^*^42.2 (23.60–85.55) multiple aneurysm	73
7	6	M	3.3 (1.71)	6.2–10.3 (10.85–21.42)		3.5–10.1 (2.76–18.45)	25		Middle fusiform aneurysm, maximum diameter 11 (23.23)		Full-range fusiform dilatation, the widest 10 (17.71) (small mural thrombus)	25
8	7	M	4.2 (3.67)	10.8 (22.34) fusiform dilatation	2.5	10.6 (18.71) mural thrombus	79		Proximal fusiform dilatation, 10.1 (20.55)		Fusiform dilatation, maximum diameter 11 (20.09), distal artery irregularly dilated, part of thrombus	79
9	6	F	2.9 (0.11)			3.9–11 (2.71–18.73) local aneurysm	1,862	3 (0.34)	3 (1.96)	1.8	Proximal 3.8 (2.71), local fusiform dilatation in the middle 11.1 (18.94) mural thrombus	1,861
10	4	F	4.4–4.7 (5.26–6.02) thrombus			2.3 (0.62)	364	4.2 (4.76)				366
11	5	M	3.1–4.5 (1.80–5.29)			4.7–14.2 (6.26–29.18) local aneurysm, multiple mural thrombus	882	Local dilated			Proximal aneurysm 17^*^12 (35.93–23.87)	885
12	7	F	4.0–11.0 (3–19.28) 7^*^3 thrombus			3.5–4.2 (2.42–3.99) strong ECHO	2,611	Aneurysm 16^*^12 (30.9–21.60)	Originated from aneurysms	Originated from aneurysms	Proximal larger aneurysms, with Calcification and thrombus in it	2,614
13	9	M	4.2 (3.17)	2.9 (1.80)	2.5	2.9–3.4 (0.85–1.94)	515				Vessel thickening from proximal 2 cm to distal, thrombus	515
14	5	M	2.3 (−0.98)			2.0 (−0.96)	1,185	3.5 (1.8)	Proximal aneurysm 6.7^*^5 (11.53–7.27) mural calcification	2.8	Middle aneurysm 9.7 (16.69) with calcification and thrombus	1,187

## Discussion

### Advantage and Limitation of ECHO

As for the means of examination, the advantage of ECHO is that it is non-invasive, convenient, and repeatable (13–15). The limitation of ECHO includes (i) that because the heart is spherical and surrounded by lungs, ECHO only examines the internal structure of the heart and provides limited evaluation on distal vessels, left circumflex artery, and a poor acoustic window in growing children (16); and (ii) for which the diagnosis of coronary artery calcification and stenosis is frequently missed when using ECHO (17). The sensitivity of ECHO in identifying coronary tumor is 66.7% (18). Furthermore, it is subject to several fallacies and is operator dependent (16). We have screened patients for CAL and thrombus using ECHO at least twice and then performed CTCA within 3 days of ECHO test for missed aneurysm and thrombus. Case 1: The patient had a history of leukemia in the past. Doctors at local hospital suspected that leukemia recurred in the early stage of his fever. Because he had rashes and conjunctival congestion, he was considered for differentiated diagnosis for KD. ECHO was performed at 5 days of illness and showed normal coronary arteries but an enlarged right ventricular and a little pericardial effusion. Then he was not diagnosed with KD and treated with methylprednisolone intravenously from the 5th to 15th day of illness. The fever subsided at 14 days after the disease onset. ECHO was performed in our hospital at 16 days of illness. Giant CAAs were found in bilateral coronary arteries, and thrombus was identified in LAD. Because the thrombus density was lower, there was still blood flowing through the coronary artery. He did not have palpitation, chest tightness, or chest pain symptoms. ECG did not show change in ST-T. However, the left ventricular end diastolic diameter (LVED) was increased to 42 (normal <36 mm). The filling defect (thrombosis) was not detected by CTCA (Figure 1). Therefore, subsequent anticoagulant therapy was applied, and the thrombus diminished on the 26th day of illness, shown by ECHO performed every other day. At the 85th day of illness, the thrombus reappeared. After thrombolytic therapy, the thrombus diminished. Our results are similar to those reported before (19); most acute coronary thrombosis in KD occurred in LAD. KD patients with coronary artery thrombosis are at risk of sudden death due to myocardial infarction.

### Advantage and Limitation of CTCA

CTCA examines the lumen structure by injecting contrast agent into the coronary artery. This method makes it possible to delineate the coronary artery anatomy with higher temporal resolution and motion-free images at all heart rates with acceptable radiation exposure (19). CTCA can identify the coronary artery calcification (20) and help visualize distal coronary artery (18). Furthermore, CTCA is a useful imaging method for delineating coronary artery in KD patients for long term follow-up, especially in older children with thick chest walls and poor acoustic windows (21). CTCA allows comprehensive evaluation of coronary arteries in children with KD (22). When KD patients with CAA developed chest tightness, chest pain, shortness of breath, edema, and oliguria, clinicians should seriously suspect that patients may have myocardial infarction, even ECHO results are normal. We highly recommend to perform CTCA for these patients. For instance, the case 2 patient was diagnosed with KD and CAL. After taking medicine for 1 month, there was no significant changes observed in the coronary artery, and the patient did not complain any discomforts. His parents stopped his medicine. Six months later, the patient could not lie on his back and had irritative cough, accompanied with edema and oliguria. The level of cTnI was significantly increased by 1.406 (normal value <0.04 μg/L), and the level of Hs-cTnT was 6.15 (normal value <0.014 μg/ml). Although there was no abnormal coronary artery blood flow in both ECHO tests, ECHO identified significantly weakened and uncoordinated motion in the ventricular wall. The left ventricular ejection fraction (LVEF) was significantly reduced to 25%. ECG showed obvious myocardial ischemia (Figure 2a). CTCA confirmed RCA occlusion caused by distal thrombosis in RCA and the stenosis at the opening of LCX. The patient's symptoms were relieved by treatment with heparin, warfarin, Plavix, and aspirin, LVEF was increased to 53%, NT Pro-BNP level was decreased, and myocardial ischemia was relieved. The anticoagulant therapy was effective in recovering myocardial blood supply. Due to consideration of decreased kidney function, CTCA was not reperformed at that time. He was transferred to Beijing children's hospital. He was intermittently monitored DIC for two times, (INR 1.1 and 1.2), platelet aggregation function was not monitored, and RCA was still blocked by CTCA 6 months later (Figure 2c). After that, his LVED increased continuously, and LVEF was about 45%. As a result, the patient was not permitted to run. Thus, CTCA can find coronary artery stenosis and distal thrombosis.

**Figure 4:**
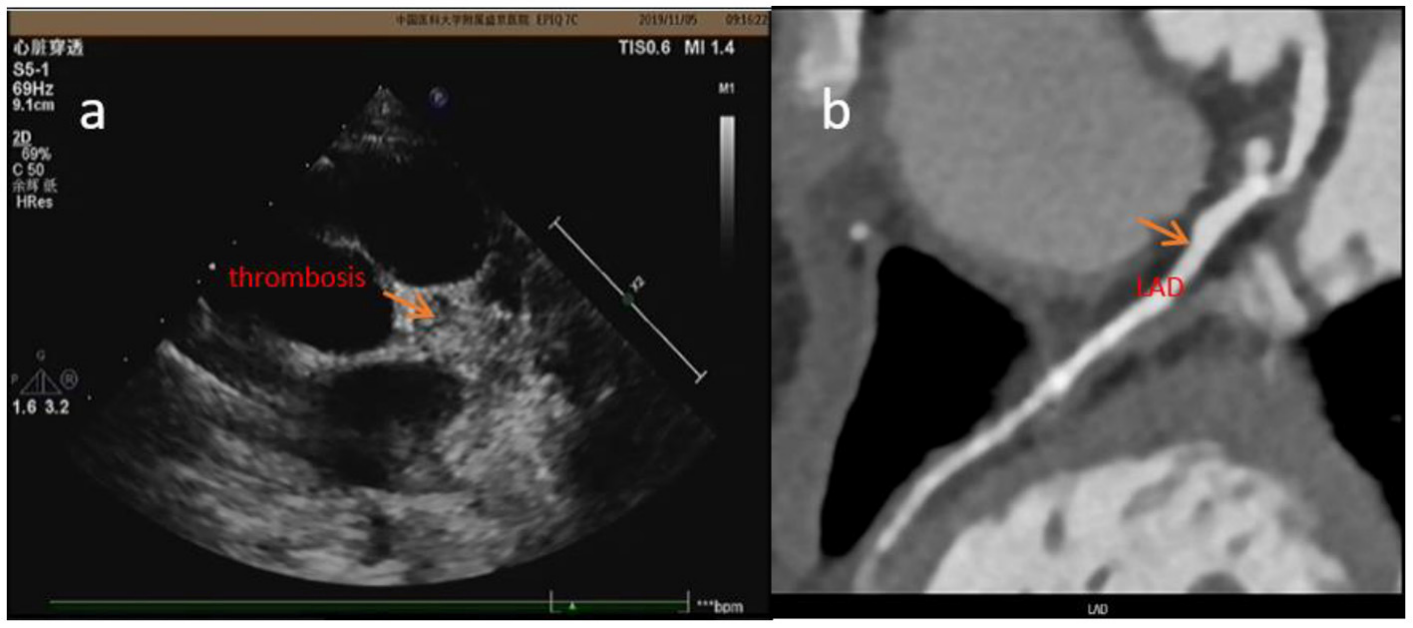
In case 4, the thrombosis was detected by ECHO **(a)** 2 days before CTCA but wasn't detected by CTCA **(b)** at about 6.5 years of illness.

Performing CTCA requires more procedural preps than performing ECHO: (i) use of an oral beta blocker to ensure heart rate <90 bpm; (ii) fasting for more than 4 h to prevent vomiting and aspiration (contrast agent injection may result in nausea); (iii) keeping the patient stable during examination (KD mostly occurs in children under 5 years old, and parents sometimes do not accept dual sedation required for performing examination); (iv) parents' concern about their children being exposed to X-rays; and (v) application of contrast agent. KD mostly occurs in allergic children (23), and they may also be allergic to iodine and have a potential risk of anaphylactic shock. Repeated use of contrast agent in a short period of time may cause renal damage (24). In addition, the requirements to perform cardiac MRA are higher than CTCA—the roaring noise of the instrument affects the sedative effect, and the examination time is longer than 30 min, which requires prolonged sedation. It also requires the application of radiation and contrast agents. Therefore, it is not frequently used to test for CAA or thrombosis but to test for ischemic cardiomyopathy (such as in case 2). Coronary artery thrombosis is the most serious complication in KD, because it can lead to myocardial infarction, coronary cardiomyopathy, and even sudden death (25) (case 3 was found to have coronary artery thrombosis and ischemic cardiomyopathy. About 2 years later, the patient suddenly died). So timely diagnosis, regular follow-up, and timely treatment are the keys to preventing disease progression. In case 4, the patient was 18 months old, and he was diagnosed with KD in our hospital on the 12th day of illness. Although he was treated with IVIG immediately, he still developed a giant CAA in LM. Because he had allergic rhinitis and used to pick his nose, after taking warfarin, dipyridamole, and aspirin, he often happened to have epistaxis and had nose frequent bleeding. His parents had to discontinue the drug. During DIC monitoring, his INR was always <2 (the standard INR was 2–3 during warfarin treatment). About 5.5 years later, there was no thrombus reported in CAA. However, at 6.5 years of illness, there was a large thrombus detected in LM. After intravenous treatment with heparin for 1 week, followed by warfarin, the thrombus did not shrink significantly (Figure 4). At 7.5 years of illness, the patient experienced chest tightness and palpitation after exercise. Prognosis in the long run was not clear. Therefore, it is important to identify thrombus timely and it is essential to execute standardized treatment.

The prognosis and quality of life in KD children are related to blood flow in coronary artery. Only stenosis of the coronary artery is rare. The most common event is the occlusion of blood flow after thrombosis in CAA. In our center, because of the lessons of the past, if patients with CAL last over 1 month, even in the absence of myocardial ischemia and with normal ECG, routine CTCA examination is required before stopping the medication (7), in order to find the CAA on the far side. In those with a giant CAA, once thrombosis was found in ECHO, CTCA should be performed in order to find whether another one was missed and determine the degree of ischemia (case 4). For those CAA patients with myocardial ischemic symptoms, even though ECHO presented normal results, CTCA must be done to in case that a distal thrombus (such as in case 2) is missed in ECHO.

In summary, ECHO is a simple, non-invasive and repeatable examination method. At present, it is still the first choice for detecting coronary artery lesions in KD. It can identify the thrombus at the proximal end of coronary artery and even with low density. However, when patients experience chest pain, sweating, fast heartbeat, elevated blood pressure and not able to lie down, combined with laboratory examination, elevated troponin, and brain natriuretic peptide, ST-T change in ECG and other symptoms of myocardial infarction, clinicians should seriously suspect coronary artery thrombosis. If ECHO does not support the diagnosis, CTCA examination must be performed to identify distal lesions (26). CTCA is considered the gold standard for detecting coronary artery lesions with high resolution and sensitivity on distal vessels, and the positive detection rate of thrombosis in our center is higher than that by ECHO. However, it comes with certain risks due to invasive procedures and the usage of contrast agent (27, 28).

Therefore, both echo and CTCA have advantages and disadvantages. They can be combined to improve the diagnosis rate for coronary thrombosis.

## Data Availability Statement

The raw data supporting the conclusions of this article will be made available by the authors, without undue reservation.

## Ethics Statement

The studies involving human participants were reviewed and approved by Shengjing Hospital, China Medical University, China. Written informed consent from the participants' legal guardian/next of kin was not required to participate in this study in accordance with the national legislation and the institutional requirements.

## Author Contributions

Y-mX: case observation, data collection and analysis, and manuscript editing. Y-qC: case observation and data analysis. X-mL, CW, RC, Y-lX, X-xY, LS, and X-zC: case observation. Q-mM: CTCA image analysis. X-nY: ECHO image analysis. X-yY: some patient diagnosis and treatment. HW: diagnosis of most patients, treatment, all image selection, data analysis, editing the discussion, and conclusion of the manuscript. All authors contributed to the article and approved the submitted version.

## Conflict of Interest

The authors declare that the research was conducted in the absence of any commercial or financial relationships that could be construed as a potential conflict of interest.
